# Case report: Concurrent malignant triton tumor and relapsed pituitary adenoma in the sellar region

**DOI:** 10.3389/fsurg.2022.1080286

**Published:** 2023-01-13

**Authors:** Jinchao Wang, Zhigang Yao, Shangchen Xu, Bin Liu

**Affiliations:** ^1^ Department of Neurosurgery, Shandong Provincial Hospital Affiliated to Shandong First Medical University, Shandong First Medical University, Jinan, China; ^2^ Graduated School of Medicine, Shandong First Medical University, Jinan, China; ^3^ Department of Pathology, Shandong Provincial Hospital Affiliated to Shandong First Medical University, Shandong First Medical University, Jinan, China; ^4^ Department of Critical Care Medicine, Shandong Provincial Hospital Affiliated to Shandong First Medical University, Shandong First Medical University, Jinan, China

**Keywords:** malignant triton tumors, sellar region, radiation, concurrent tumor, relapsed pituitary adenoma

## Abstract

Malignant triton tumor (MTT) is a rare kind of malignant peripheral nerve sheath tumors, histologically characterized by rhabdomyoblastic differentiation. There are limited reports of MTT occurring in the intracranial area. The treatment modality consisting of total surgical resection plus post-operative radiotherapy is generally accepted. However, even with optimal treatment, most patients will die within a few months. We report a 71-year-old man with a history of pituitary adenoma, who underwent surgical treatment and postoperative gamma knife therapy. Magnetic resonance imaging (MRI) of the brain revealed a mass with two distinctive components in the sellar area. Postoperative pathology found that the lesion consisted of a MTT and a relapsed pituitary adenoma. The present case is the first report of MTT that occurred in the sellar area. It is also the first case of intracranial MTT with other concurrent tumors (relapsed pituitary tumors). Meanwhile, this case has a clear history of radiation therapy, suggesting that the occurrence of MTT may be related to radiation.

## Introduction

MTT is a malignant peripheral nerve sheath tumor (MPNST) with rhabdomyoblastic differentiation. In 1938, Masson and Martin first described this compound tumor. They proposed that this tumor induces skeletal muscle differentiation in the same way as normal nerves (1). It got the name as malignant salamander tumor in 1973, basing on previous observations that if the sciatic nerve was implanted into the dorsal side, the salamander would produce redundant limbs composed of striated muscle, bone and nerve tissue. They mimic other brain tumors on MRI, which brings challenges to preoperative diagnosis. This the first MTT case reported with concomitant pituitary adenoma in the sellar area.

## Case report

### History and presentation

**Figure 1:**
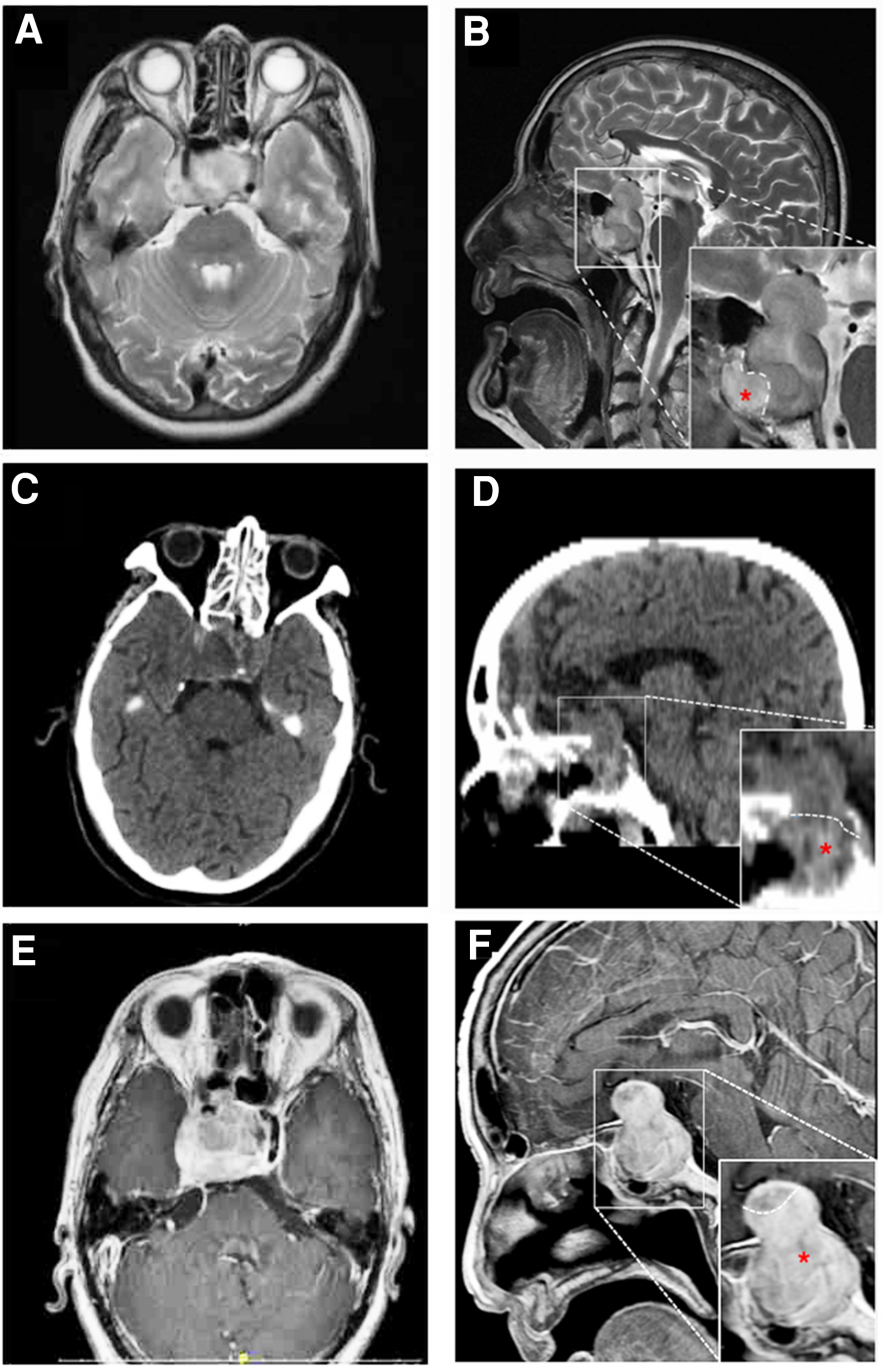
MRI (**A,B,E,F**) and CT (**C,D**) findings in a patient with pituitary adenoma (PA) and concurrent malignant triton tumor (MTT) in sellar area. On 20 days before the operation, axial image (**A**) and saggittal (**B**) T2-weighted images revealed that the lesion is consisted of two distinctive components with a clear border. The upper and lower components were histologically diagnosed as pituitary adenoma and MTT (red asterisk), respectively. Four days after the operation, axial (**C**) and saggittal (**D**) CT images showed that MTT component was totally removed, while residual PA could be seen in suprasellar area. Fifty-six days after the operation, axial (**E**) and saggittal (**F**) contrast-enhanced MR images revealed that MTT has recurred.

A 71-year-old man was referred to our hospital with impaired vision and intermittent nausea and vomiting in August 2020. He had a history of nausea and vomiting in June 2010. MRI revealed a lesion in the sellar region in a local hospital. At that time, he immediately underwent gross total surgical resection and pathological examination confirmed the lesion as a pituitary adenoma. Subsequently, he underwent adjuvant gamma knife treatment in September 2010. Thereafter, the symptoms disappeared, except that he experienced hyponatremia several times without formal treatment. In July 2020, the patient experienced vision loss, recurred intermittent nausea, and vomiting. The local hospital diagnosed cataract and a cataract surgery was performed accordingly. After the cataract surgery, the vision recovery was not ideal and continued to worsen. Meanwhile, intermittent nausea and vomiting did not alleviate. Intracranial MRI revealed an irregular mass with 2 distinctive components in sellar area (Figures 1A,B). An endocrine panel demonstrated an elevated serum follicle-stimulating hormone (FSH) level. Combined with medical history, it was considered that the tumor recurred. The patient was then referred to our hospital and underwent partial resection of the lesion with a transnasal transsphenoidal approach.

**Figure 2:**
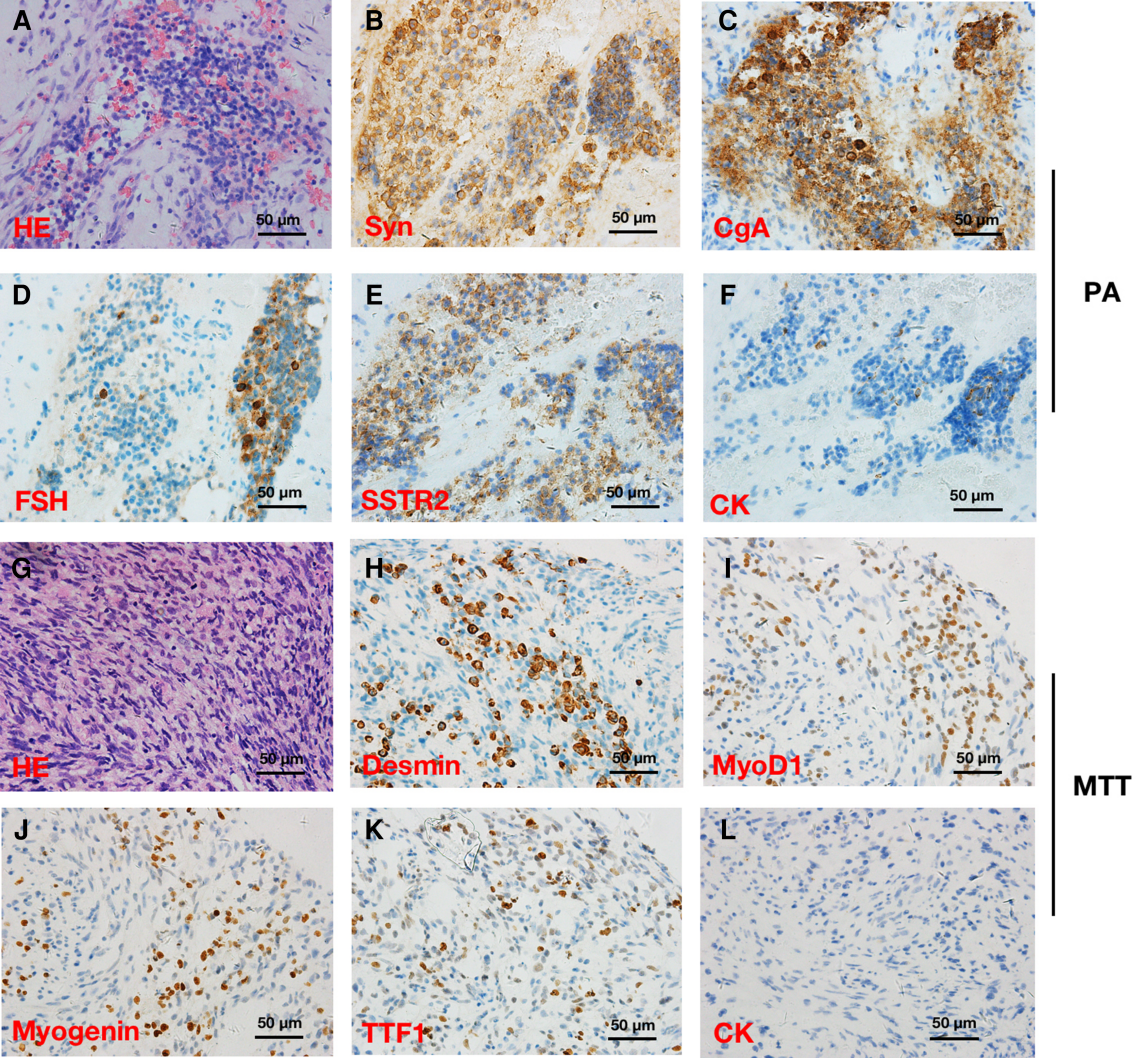
Hematoxylin and eosin (HE) and immunohistochemical stainings in a patient with pituitary adenoma (PA) (**A–F**) and concurrent intracraninal malignant triton tumor (MTT) (×400) (**G–L**). HE (**A**) and immunoreactivity of synaptophysin (Syn) (**B**), chromogranin A (CgA) (**C**), follicle-stimulating hormone (FSH) (**D**), somatostatin receptor 2 (SSTR2) (**E**), and cytokeratin (CK) (**F**) supported the diagnosis of PA. Round and fussiform cells with eosinophilic cytoplasm morphologically consistent with rhabdoid differentiation were observed in HE staining (**G**). Desmin (**H**), Myogenin (**I**), and myogenic differentiation 1 (MyoD1) (**J**) immunoreactivity supported the diagnosis of a MTT. Notably, TTF-1 (**K**) was immunopositive, while CK (**L**) was immunonegative in the MTT.

Pathological findings revealed that the tumor was composed of a pituitary adenoma (Figures 2A–F) in the upper area and a concurrent MTT in the lower area with a clear border (Figures 2G–L). In the pituitary adenoma component, immunostaining was positive for synaptophysin (Syn), chromogranin A (CgA), FSH, cytokeratin (CK), and somatostatin receptor 2 (SSTR2). In the MTT component, immunostaining was positive for Desmin, Myogenin, thyroid transcription factor-1 (TTF-1), and myogenin differentiation 1 (MyoD1). Interestingly, TTF-1, which could be found in 66.7% of schwannomas (2) and is also a novel marker of pituitary tumors of the posterior lobe (3, 4), was immunoreactive (Figures 2H). CK was immunonegative in the MTT portion. In addition, the average Ki-67 staining (MIB labeling index) in the PA and TT portions were 3% and 40%, respectively.

On the fourth day after the operation, computer tomography (CT) examination of the brain revealed that the tumor mass containing MTT component in sphenoidal sinus was gross-totally resected, while the pituitary adenoma was partially resected with residual tumor in the suprasellar area (Figures 1C,D). Post-operatively, the patient developed hyponatremia and recovered after active treatment. The patient's vision loss also recovered well after the operation. However, blurred vision recurred and continued to worsen 7 days after the operation. A re-examination of cranial MRI 56 days after the operation showed that the residual tumor had grown again (Figures 1E,F). The patient was blind and refused to undergo further surgical treatment and radiotherapy. Eventually, he died 64 days after the second operation.

## Discussion

MTTs is a subgroup of malignant peripheral nerve sheath tumors (MPNST), which are reported to exhibit rhabdomyosarcoma-like differentiation and follow a particularly aggressive course (5). Due to the rarity of MTT, merely eleven intracranial cases have been reported, which is summarized in Table 1. To the best of our knowledge, the present case is the first report of MTT in sellar region with a clear history of radiation and a concurrent relapsed pituitary adenoma.

**Table 1:** Summary of reports of intracranial malignant triton tumors (MTTs).

	Age (y)	Sex	NF-1	Previous radiation	Site of MTT	Concurrent other tumors	Myoglobin or Desmin	Management	Survival time
Best, 1987	24	F	No	No	Cerebellopontine angle	No	Yes	Surgery	4 months
Han et al., 1992	47	F	No	No	Cerebellopontine angle	No	Yes	Surgery	10 months
Comey et al., 1998	44	M	No	Yes	Cerebellopontine angle	No	Yes	Surgery, gamma knife, radiotherapy	6.5 years
De Cauwer et al., 2000	57	M	Yes	No	Lateral ventricle	No	Yes	Surgery, chemotherapy, radiotherapy	5 months
Bornstein-Quevedo et al., 2003	3	M	No	No	Parietooccipital	No	Yes	Surgery	10 days
De Cauwer et al., 2007	57	M	No	No	Cerebellopontine angle	No	Yes	Surgery	5 months
Lau et al., 2010	42	M	No	No	Nasal cavity/cribriform plate	No	Yes	Endoscopic biopsy	Not available
Gong et al., 2012	55	F	No	No	Cerebellopontine angle	No	Yes	Surgery	Not available
Smith et al., 2014	26	M	Yes	No	Bifrontal	No	Yes	Surgery, radiotherapy	Shortly after surgery
Eros et al., 2018	74	F	No	Yes	Middle cranial fossa	No	Yes	Surgery, chemotherapy, radiotherapy	4 months
Adriano et al., 2019	5	F	Yes	unknown	Right frontal	No	Yes	Surgery	Not mentioned
The present case	71	M	Yes	Yes	Sellar region	Yes	Yes	Surgery	64 days

It is generally believed that the striated muscle cells in tumors are induced by or directly transformed from Schwann cells (6). In the present case, the visual acuity of the patient recovered completely after operation and the MTT component located apart from the optic nerve, which can basically exclude the origination of MTT from the optic nerve sheath.

MTTs mimic gliomas on MRI (7), where both of them show irregular masses, fuzzy boundaries, a peripheral increase, a low or equal signal on T1, and a high signal on T2 images (8). Genomic and proteomic studies have begun to reveal the complexity of chromosome and transcriptional changes behind these tumors, but reliable cytogenetic markers have not been established to distinguish MTT and MPNST and/or explain the obvious differences in their behavior (9–11). Daimaru et al. proposed a relatively widely accepted definition, including the following (12): (1) The first criterion is that, it is shown as a malignant schwannoma under the microscope and contains focal rhabdomyoblasts; The second criterion is that, the tumor is mainly composed of striated muscle and focal Schwann cells, which are found in neuropathy or NF-1 expression. Now, the diagnosis of MTT commonly depended on these criteria and on immunohistochemical findings (13).

The differential diagnosis of MTT includes other spindle cell malignant tumors, such as fibrosarcoma, synovial monophase sarcoma, leiomyosarcoma, low-grade fibroblast sarcoma, especially spindle cell rhabdomyosarcoma (14). Low-grade myofibroblastic sarcomas, synovial sarcomas, and fibrosarcomas do not contain rhabdomyoblasts and do not respond to skeletal muscle staining such as Desmin. Rhabdomyosarcoma, especially embryonic rhabdomyosarcoma, is mainly consisted of undifferentiated round cells and spindle cells (15). However, the composition of rhabdomyosarcoma is different from that of MTT, and the mean age of patients with rhabdomyosarcoma is younger than the patients with MTTs. In addition, there are usually high-density cell areas surrounding blood vessels, alternating with parvicellular areas rich in mucosal intercellular material.

In the present case, immunostainings were positive for skeletal muscle markers including Myogenin, Desmin, and Myod1, which supports the diagnosis. Interestingly, TTF-1 immunoreactivity was observed in the MTT component. Given that TTF-1 could be found in 66.7% of schwannomas (2), this result also supported the diagnosis of MTT. Moreover, in the 2017 WHO classification of pituitary tumors (4), TTF-1 expressing pituitary tumors of the posterior lobe represent a morphological spectrum of a single nosological entity. Considering the location of the tumor in our present case, we boldly speculate that there is possibility that MTT portion might arise from the mesodermal components in the posterior lobe of pituitary gland. Additionally, S100 protein is immunopositive in 50%–90% of MPNSTs (13), while S100 protein was negative in our case. We hypothesize that it might be explained by the following reasons: (1) the MTT tissue in our present case was not fully differentiated to express S100; (2) the S100-positive tissue was discarded or vacuumed during the surgical operation and was not provided for pathological examination.

It is reported that 8% of patients with MTT had been exposed to radiation, which is recognized as a risk factor (16–18). In addition, even therapeutic radiation can trigger the formation of MTT (19). Our report also provides evidence that the occurrence of MTT might be induced by radiation therapy.

Since MTT is rare and lack of prospective clinical trials, there is no standard treatment available. The 5-year survival rate of patients with MTT was 11%–14%, which was significantly lower than those patients with MPNST (34%–52%) (13, 14, 16). The adverse prognostic factors of MTTS mainly include NF-1, age, size, surgical method, positive surgical margin, early local recurrence, and the location of the tumor in the trunk (19).

Complete resection can improve the prognosis, reduce the risk of local recurrence and metastasis, and improve the efficacy of adjuvant therapy. In contrast, subtotal cytoreductive surgery cannot improve the survival rate of MTT patients (13). Adjuvant radiation therapy reduces the risk of death, but is not associated with a reduction in progression or recurrence (16). Although adjuvant chemotherapy has not been proved to be effective (16), there are certain evidence that patients who respond to neoadjuvant chemotherapy should also receive adjuvant therapy immediately (20). However, most MTT patients usually die within a few months, even after receiving optimal treatments (18).

In the future, we hope to have more cases and studies to better understand this tumor and provide more reliable diagnostic methods, especially pathological diagnostic criteria and more effective treatment strategies.

## Conclusion

In summary, to our knowledge, this is the first report of MTT in sellar region with concurrent other tumors. This case had a history of radiation exposure after the first operation, which suggests that the occurrence of MTT might be caused by radiation. The patient received partial resection of the lesion with a transnasal transsphenoidal approach. The patient did not receive any adjuvant treatment postoperatively. Eventually, he died 64 days after the operation. Unfortunately, due to the rapid progress of the patient's condition, this case did not have much value for reference in treatment. In the future, we hope to have more cases and studies to better understand this tumor and provide more reliable diagnostic methods, especially pathological diagnostic criteria and more effective treatment strategies.

## Data Availability

The original contributions presented in the study are included in the article/Supplementary Material, further inquiries can be directed to the corresponding authors.
